# Tracemetals, neuromelanin and neurodegeneration: An interesting area for research

**DOI:** 10.4103/0019-5545.37310

**Published:** 2007

**Authors:** T. S. Sathyanarayana Rao, Luigi Zecca, K. Jagannatha Rao

**Affiliations:** Department of Psychiatry, JSS Medical College Hospital, Ramanuja Road, Mysore - 570 004, India; * Institute of Biomedical Technologies, Italian National Research Council, 20090 Segrate, Milan, Italy; ** Department of Biochemistry, Central Food and Technological Research Institute, Mysore, India

## INTERNATIONAL SCENARIO

The understanding of cellular neurodegeneration in brain disease is today's major challenge. The mapping of region-specific changes in the brain during neurodegeneration is a thrust area in neuroscience. Bipolar disorders are classical neuropsychiatric disorders where crosstalk between environmental factors and genetics plays an important role in the etiology of these disorders. Locus coeruleus (LC) is the main brain region containing norepinephrine neurons. The rostral projections from these neurons seem to be involved in the modulation of neuronal activity, metabolism and memory[1,2] whereas the spinal cord projections are known to modulate spinal motoneuron function.[3,4] Neuronal loss in LC occurs in Parkinson's disease (PD) and Alzheimer's disease and Down's syndrome[5,6] in the midbrain regions. In Alzheimer's disease and Down's syndrome, it is not clear whether neuronal loss in LC is a primary event or a consequence of retrograde degeneration of cortically projecting cells due to the loss of cortical synapses. In PD, results have showed neuronal loss in substania nigra (SN) compared to LC.[5–8] However, a recent study reported extensive impairment of LC neurons in PD.[9] SN and LC share anatomical and biochemical similarities, both being pigmented because of neuromelanin (NM) and both composed of catecholaminergic neurons. The relationship of factors like iron, ferritins and NM[10–14] to aging has been analyzed in previous studies. These factors may influence neuronal viability and undergo dramatic changes in PD. As aging is an important risk factor for PD, it would be relevant to establish the age-related changes in elements involved in oxidative stress like iron, copper and related molecules, all of which have been implicated as putative pathogenic factors. Due to their role in peroxidation, changes in iron, copper, their major storage proteins ferritin and ceruloplasmin (CP), together with the enzymes manganese-superoxide dismutase (SOD) and copper/zinc-SOD were determined in human SN and LC at various ages. Being a strong chelator of iron and other toxic metals,[14] NM has been shown to be a strong modulator of their cellular effects.[15–17]

Luigi from the Italian group did a comparative analysis of metal-related neuronal vulnerability in two brainstem nuclei, the locus coeruleus (LC) and substantia nigra (SN), which are known targets of the etiological noxae in PD and related disorders. LC and SN pars compacta neurons both degenerate in PD and other Parkinsonisms although LC neurons are comparatively less affected with a variable degree of involvement. In this study, iron, copper and their major molecular forms like ferritins, ceruloplasmin, neuromelanin (NM), manganese-superoxide dismutase (SOD) and copper/zinc-SOD were measured in LC and SN of normal subjects at different ages. Iron content in LC was much lower than that in SN and the ratio of heavy chain ferritin / iron in LC was higher than in SN. The NM concentrations were similar in LC and SN although the iron content in NM of LC was much lower than in SN. In both regions, heavy and light chain ferritins were present only in glia and were not detectable in neurons. This data suggests that iron mobilization and toxicity are lower in LC neurons than in SN and are efficiently buffered by NM. The greater damage occuring in SN could be related to the higher content of iron. Ferritins accomplish the same function of buffering iron in glial cells. Ceruloplasmin levels were similar in LC and SN although copper content was higher in LC. However, the copper content in NM of LC was higher than that of SN, indicating a higher copper mobilization in LC neurons. Manganese-SOD and copper / zinc-SOD had similar age-related trends in LC and SN. These results may explain some of the underlying lower vulnerability of LC compared to SN in Parkinsonian syndromes.[18]

Trace elements play a dual role in the biological system through their interaction with biomolecules. They regulate a number of cellular metabolic reactions while a few of them act as etiological agents in many environmentally induced neurological disorders.[19-20] Trace elements are required at optimum concentration for the proper functioning of the human biological system. Their deficiency would cause various metabolic disorders while their high levels are toxic[3]. Non-essential elements like aluminium, lead and cadmium are highly toxic even in trace amounts and reported to be associated with neurological disorders. Studies have shown that trace elements are involved in neuropsychiatric illness.[21] Studies have shown that copper, zinc and cesium deficiencies are seen in women affected by chronic depression.[22–23] It has been found that the levels of magnesium and zinc are decreased in serum samples of schizophrenia and dementia patients.[24] Although pivotal biochemical alterations underlying the neuropsychiatric disorders are unknown, changes in trace elements play an important role in bipolar disorders. Recently, essential elements like vanadium have been implicated as a causative factor for bipolar mood disorder while the elevation of vanadium and molybdenum levels has been reported in serum samples of bipolar mood disorder patients. Christiansen *et al*.[25] have shown that lithium alters the levels of elements such as calcium and magnesium in serum samples during treatment. This data is only part of the limited data set available on the concentration of trace elements in the serum of individuals with bipolar mood disorders while there are no interelemental complexity studies in the literature pertaining to trace elemental levels in serum samples of patients suffering from three types of bipolar disorder. Furthermore, while the above-mentioned studies have focused on individual elements, no attempt has been made to understand the interelemental relationships and trace elemental homeostasis in bipolar disorder.

We assessed the serum levels of eleven elements in bipolar disorders types I, II and V and attempted to understand the complexity of trace elemental interrelationships in bipolar disorders as compared to the control group. Trace elements, namely, Na, K, S, Ca, Mg, P, Cu, Fe, Zn, Mn and Al were analyzed in serum samples of Bipolar I (*n* = 40), Bipolar II hypomania (*n* = 25), Bipolar II depression (*n* = 25), Bipolar V depressives (*n* = 25) and control (*n* = 25) using Inductively Coupled Plasma-Atomic Emission Spectrometry (ICP-AES). The patients were assessed as per the standard diagnostic criteria of DSM IV and classified into types I, II and V by a psychiatrist using the concept of Young and Klerman. The significant results were a) in Bipolar I (Mania), Na, K, P, Cu, Al and Mn were elevated significantly (*P*<0.001); b) in Bipolar II hypomania, Na, S, Al and Mn were increased significantly (*P*<0.02) while in Bipolar II depression, Na, K, Cu and Al were increased significantly (*P*<0.001); c) in Bipolar V, Na, Mg, P, Cu and Al were increased significantly (*P*<0.002) but S (*P*<0.00001) (Comment: but this was increased in b), Fe (*P*<0.002) and Zn (*P*<0.004) were decreased in all three bipolar groups. The data revealed disturbance in the charge distribution and interelemental interdependency in bipolar group serum compared to that of the control group. These results suggest that there is definite imbalance in trace elemental homeostasis as evidenced by trace elemental interrelationships in serum samples of bipolar groups compared to those of the control group.[26–28]

Based on our new and others' findings and Dr. Zecca's highly significant contributions, we developed a hypothetical model explaining the possible relevance of trace elemental homeostatic imbalance in the serum of bipolar disorder to their effects in brain.

With these findings, the following two pathways can be deduced. In pathway I, increased Al levels in the serum of bipolar disorder patients is likely to alter the trace elemental homeostasis pool. Irrespective of whether elements are primary risk factors or consequences of disease processes, a change in an individual metal ion will upset the elemental homeostasis pool resulting in significant imbalance in elemental levels and charge distribution pattern in the body system. The element-to-element mole ratios of Al / Fe and Al / Zn increased because of high concentrations of Al present, which alters other elemental levels. This is evidenced by the existence of an inverse relationship between Al and S.

The elevated Al levels may disturb the metal homeostatis in the serum by increasing the levels of paramagnetic oxidant elements like Cu and Mn and decrease the levels of Zn, which is an antioxidant metal (required for the production of Cu Zn-SOD and Zn-thionein) essential to prevent oxidative damage. Elevated Al level is found to increase superoxide dismutase (SOD) activity to protect the cell from oxidative damage. Recently, Kolugulu *et al*.[29] showed SOD activity levels are higher in bipolar disorder patients' serum and also reported the presence of lipid peroxidation. Increased Al levels may be one of the reasons for high SOD activity in bipolar disorder. Elevated levels of paramagnetic elements Cu and Mn in serum may catalyze the conversion of H_2_ O_2_ to potent hydroxyl radicals, which could lead to oxidative damage.

In pathway 2, the elements in the serum possibly reflect the brain elemental homeostatis. Previous studies demonstrated that Al and Fe levels decreased in cerebrospinal fluid (CSF) while they are elevated in the brain. Al is known to be cotransported with the Fe-Transferrin complex in neurological disorders. In the normal brain, Fe and Al compete for transport across the blood brain barrier and Al can cross the blood brain barrier with the help of ferritin. Al can promote Fe-mediated oxidative stress by inhibiting catalase activity in the brain and also by causing mitochondrial dysfunction leading to oxidative stress and neuron dysfunction. These pathways highlight the fact that trace elemental imbalances in bipolar disorder patients' serum may cause imbalances in trace elemental levels in the brain which may lead to oxidative stress and damage the biomolecules like DNA, lipids and proteins. This may be the reason for alteration in the neurotransmitter receptors and the levels of secondary messengers in bipolar disorder patients' brain. It can be inferred that elemental homeostatic imbalance results in the imbalance of biochemical events and oxidative stress in bipolar disorder, which may later manifest as neurodegeneration. There are few studies supporting our hypothesis. PET scan study by Bier *et al*.[30] reported that depression might cause neurodenegeneration. Another study by Buhl *et al*.[31] demonstrated the presence of neuritic pathology in bipolar mood disorder. On the contrary, Damadzic *et al*.[32] reported that neuritic pathology is lacking in the entorhinal cortex, subiculum and hippocampus in middle-aged adults with schizophrenia, bipolar disorder or unipolar depression. We believe that neuritic pathology may be the final onset phase of neurodegeneration with initial phases probably being neuropsychiatric phenomena with biochemical and brain function alteration. Our findings illustrate that more studies are required to link neuropsychiatry to neurodegeneration.
